# Prevalence and characteristics of and risk factors for impacted teeth with ankylosis and replacement resorption – a retrospective, 3D-radiographic assessment

**DOI:** 10.1186/s40510-024-00531-5

**Published:** 2024-08-19

**Authors:** Michael Nemec, Giacomo Garzarolli-Thurnlackh, Stefan Lettner, Hemma Nemec-Neuner, André Gahleitner, Andreas Stavropoulos, Kristina Bertl, Erwin Jonke

**Affiliations:** 1 Division of Orthodontics, University Clinic of Dentistry, Medical University of Vienna, Sensengasse 2a, Vienna, 1090 Austria; 2 Core Facility Hard Tissue and Biomaterials Research, University Clinic of Dentistry, Medical University of Vienna, Sensengasse 2a, Vienna, 1090 Austria; 3 Department of Diagnostic Radiology, Division of Osteoradiology, General Hospital, Medical University of Vienna, Spitalgasse 23, Vienna, 1090 Austria; 4 Department of Periodontology, Faculty of Odontology, University of Malmö, Carl Gustafs väg 34, Malmö, 205 06 Sweden; 5 Division of Conservative Dentistry and Periodontology, University Clinic of Dentistry, Medical University of Vienna, Sensengasse 2a, Vienna, 1090 Austria; 6 Department of Periodontology, Dental Clinic, Faculty of Medicine, Sigmund Freud University Vienna, Freudplatz 3, Vienna, 1020 Austria; 7 Department of Periodontology, Blekinge Hospital, Byggnad 13, Hälsovägen, Karlskrona, 371 41 Sweden; 8 Division of Oral Surgery, University Clinic of Dentistry, Medical University of Vienna, Sensengasse 2a, Vienna, 1090 Austria; 9 Department of Periodontology, School of Dental Medicine, University of Bern, Freiburgstrasse 7, 3010 Bern, Switzerland

**Keywords:** 3D radiographic assessment, Dental ankylosis, Predicted risk, Prevalence, Replacement resorption, Tooth impaction

## Abstract

**Background:**

Large variation in the prevalence of ankylosis and replacement resorption (ARR) is reported in the literature and most studies have relatively small patient numbers. The present retrospective study aimed to provide an overview on prevalence, location of, and associated risk factors with ARR based on a large sample of computed tomography (CT) / cone beam computed tomography (CBCT) scans of impacted teeth. The results should allow clinicians to better estimate the risk of ARR at impacted teeth.

**Methods:**

The CT/CBCT scans of 5764 patients of a single center in Central Europe were screened with predefined eligibility criteria. The following parameters were recorded for the finally included population: gender, age, tooth type/position, number of impacted teeth per patient, and presence/absence of ARR. For teeth with ARR the tooth location in reference to the dental arch, tooth angulation, and part of the tooth affected by ARR were additionally registered.

**Results:**

Altogether, 4142 patients with 7170 impacted teeth were included. ARR was diagnosed at 187 impacted teeth (2.6%) of 157 patients (3.7%); 58% of these patients were female and the number of teeth with ARR per patient ranged from 1 to 10. Depending on the tooth type the prevalence ranged from 0 (upper first premolars, lower central and lateral incisors) to 41.2% (upper first molars). ARR was detected at the crown (57.2%), root (32.1%), or at both (10.7%). After correcting for confounders, the odds for ARR significantly increased with higher age; further, incisors and first/second molars had the highest odds for ARR, while wisdom teeth had the lowest. More specifically, for 20-year-old patients the risk for ARR at impacted incisors and first/second molars ranged from 7.7 to 10.8%, but it approximately tripled to 27.3–35.5% for 40-year-old patients. In addition, female patients had significantly less often ARR at the root, while with increasing age the root was significantly more often affected by ARR than the crown.

**Conclusion:**

ARR at impacted teeth is indeed a rare event, i.e., only 2.6% of 7170 impacted teeth were ankylosed with signs of replacement resorption. On the patient level, higher age significantly increased the odds for ARR and on the tooth level, incisors and first/second molars had the highest odds for ARR, while wisdom teeth had the lowest.

## Introduction

Impacted teeth are a frequent finding potentially requiring a complex and multi-disciplinary treatment plan [1]. The etiology of tooth impaction is multifactorial including a variety of local (e.g., supernumerary teeth, odontogenic tumors, ankylosis) and systemic factors (e.g., endocrine disorders, genetic/inherited) [2]. After the third molars (24.4%), the maxillary canines are most often impacted with a prevalence of 0.8–3.3%, followed by the premolars (1.2%) [1, 3–5]. While impaction of third molars requires either no treatment or relatively straightforward surgical extraction, the treatment plan for impacted maxillary canines and mandibular premolars can be more complex and include several disciplines, i.e., in many cases surgical exposure and orthodontic traction and alignment is indicated to improve functional and aesthetic aspects for such patients [6].

In this context, the success of orthodontic alignment depends on various factors such as position (e.g., buccal, palatal, transmigrated) and inclination of the impacted tooth, presence/absence of crowding in the dentition, patient’s cooperation, etc. Further, a larger distance to the occlusal plane and/or transmigration of the impacted teeth might prolong or even imped orthodontic alignment [7, 8]. More recently, scientific interest in orthodontic tooth movement focused on the role and function of mechano-sensitive non-coding RNAs, including microRNAs and long non-coding RNAs, and their specific role in bone remodeling; these mechano-sensitive non-coding RNAs might even offer in the future therapeutic possibilities [9].

Another factor interfering with successful orthodontic alignment can be the presence of ankylosis at the impacted tooth [10]. As outlined previously [11] and also by a recent comprehensive review [12] there is still no universally accepted classification for ankylosis and the various types of tooth resorption. Ankylosis as such can occur without any resorption, i.e., ankylosis is defined as the loss of periodontal ligament space leading to a direct contact between the bone and tooth. This in turn might later lead to external replacement resorption. In addition, such an external replacement resorption process might not be limited to the root surface but extend also to the crown [13]. Impacted teeth affected by ankylosis and replacement resorption (ARR) do not respond to orthodontic traction or stop after initial movement in most of the cases [14].

The prevalence of ARR is ranging widely in the literature and naturally depends vastly on the investigated population, i.e., among impacted teeth, prevalence rates between 1 and 32% are reported [7, 13, 15–21]. The presence of ARR has been significantly associated with anterior teeth, the maxillary arch, single rooted teeth, and tooth impaction [20]. Damage of the periodontal ligament during surgical exposure of the impacted tooth increases the risk of ARR [16]. ARR often develops rapidly, i.e., within months. Therefore, the progress of orthodontic alignment should be observed on a regular base with intraoral radiographs and/or orthopantomographs (OPTGs) [14]. In case of suspicion of ARR, computed tomography (CT) or cone beam computed tomography (CBCT) is considered most reliable for confirming the diagnosis [22]. Nevertheless, access to CBCT may not be available in many parts of the world, and therefore knowledge about the approximate risk for ARR, as well as of factors being predictive of ARR could help in treatment planning.

Most of the available studies on the prevalence of and factors associated with ARR of impacted teeth have focused on maxillary canines, with relatively small sample sizes ranging from 30 to 225 and, at least partly, without 3-dimensional radiographic diagnostic [13, 15–19, 21]. The aim of the present study, based on a very large sample of CT/CBCT scans of impacted teeth, was to (1) assess the prevalence, location of, as well as possible factors associated with ARR at impacted teeth, and to (2) calculate the predicted risk for ARR at impacted teeth in different clinical scenarios. The results should allow clinicians to better estimate the risk of ARR at impacted teeth.

## Materials and methods

### Patient population and eligibility criteria

The study protocol of this retrospective radiographic study was approved by the ethics committee of the Medical University of Vienna (1405/2021) and reporting of the manuscript complies with the STROBE guidelines (Appendix 1). All patients, who received a CT or CBCT scan between 11/2012 and 07/2020 due to suspected ARR, were included (“sample I”). Additionally, 5500 out of more than 7000 patients, who during the same time received a CT or CBCT scan due to suspected pathologies and/or anatomic considerations in connection to impacted teeth, were randomly selected (“sample II”), i.e., simple random sampling without replacement from the pool of eligible patients was performed. This resulted in a total sample size of 5764 patients. The following exclusion criteria were applied on both samples (I + II): (1) lack of an OPTG, (2) no permanent tooth impaction (i.e., patients presenting only with impacted deciduous teeth, auto-transplanted teeth, and/or teeth after trauma treatment were excluded), and (3) artefacts impeding judgement of the region of interest. All following assessments have been performed by a single observer after calibration with an experienced dental radiologist.

### CT and CBCT scans

Dental CT and CBCT scans were acquired by one the following devices, using the following protocols:

- Siemens Somatom Sensation 4 (Siemens AG, Erlangen, Germany) with 2 × 0.5 mm slice thickness, 1.0 mm table feed, 1 s scan time, 120 kV, 80 mA, high-resolution bone filter, (2012–2018)
- Siemens Somatom definition AS (Siemens AG, Erlangen, Germany) with 0.5 mm slice thickness, 0.5 mm table feed, 120 kV, 140 mA, high resolution bone filter (2018–2020).
- 3D Accuitomo MCT-1 (J Morita Manufacturing Corp., Kyoto, Japan) with 0.25 mm slice thickness, 90 kV, 5 mA, high-resolution bone filter (2012–2020).

### Assessment of tooth impaction

A tooth was classified as impacted, if the crown was covered by bone in the OPTG and/or CT/CBCT scan, or regular eruption was not expected either due to tooth transmigration or angulation. Additionally, a tooth was defined as impacted, if it did not reach the occlusal plane prior to a defined age; the following age limits were used for the definition of impaction [23, 24]:

- Incisors: 8 years.
- Canines: 14 years.
- First premolars: 12 years.
- Second premolars: 13 years.
- First molars: 7 years.
- Second molars: 13 years.
- Third molars: 20 years.

### Parameters assessed in all participants

The following information was extracted for all participants: (i) gender, (ii) age at timepoint of CT/CBCT scan, and (iii) type (i.e., CT or CBCT) and (iv) region of scanning (upper or lower jaw or a specific tooth type/position). Further, the following parameters were recorded, based on OPTGs: (i) presence of tooth impaction, (ii) type/position of the impacted tooth, and (iii) number of impacted teeth per patient; and based on CT/CBCT scans: (i) type/position of the impacted tooth recorded in the CT/CBCT scan, (ii) number of impacted teeth recorded in the CT/CBCT scan, and (iii) presence/absence of ARR at the impacted tooth.

### Parameters assessed in patients with ARR

Only teeth showing signs of ARR were included herein. ARR was diagnosed for teeth where parts of the tooth were resorbed and replaced by bone in the lack of a periodontal ligament space [12]. This was previously also defined as “clear signs of ankylosis”, i.e., no visible periodontal ligament space, but visible resorption, and presence of tissue replacement [25]. Hence, teeth with only a lack of visibility of the periodontal ligament space were not included herein.

**Fig. 1:**
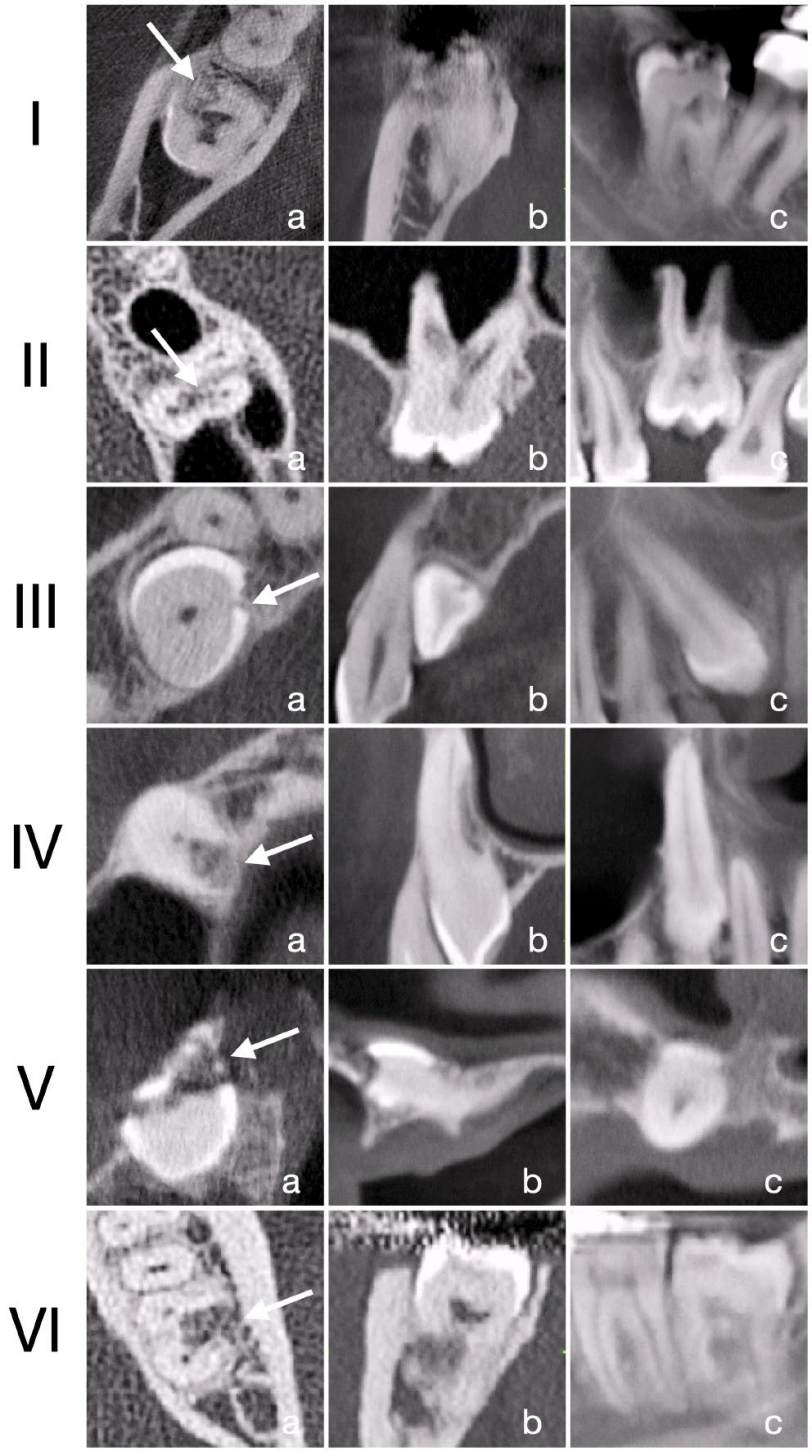
Various examples of impacted teeth with ARR (indicated by the white arrow); (a) axial, (b) coronal, and (c) sagittal view. (I) tooth #48 with ARR in the cervical third of the mesial root; (II) tooth #26 with ARR at the palatal and distobuccal root; (III) tooth #13 with mesial angulation and a minor ARR at the crown; (IV) tooth #13 with vertical angulation and ARR at the middle third of the root; (V) tooth #11 with horizontal angulation and ARR at the crown; and (VI) tooth #38 with ARR in the furcation area

In patients with at least one impacted tooth with ARR (Fig. 1), the following parameters were recorded for the teeth with ARR, based on CT/CBCT scans: (i) location of the tooth in reference to the dental arch (i.e., central, buccal, or lingual/palatal), (ii) angulation of the tooth (i.e., vertical, mesioangular, distoangular, buccal, palatal/lingual, or horizontal), (iii) part of the tooth affected by ARR (i.e., dental crown or root), and (iv) part of the root affected by ARR (i.e., furcation area, entire root, or cervical, middle or apical third of the root).

### Sample size calculation

Based on the formula for binomial confidence intervals and assuming a worst-case scenario of 50% of patients with ARR, we calculated that in total at least 4269 patients are needed to achieve a width of 0.03 for the resulting interval. To achieve this sample size after applying eligibility criteria, 5500 patients were – in addition to sample I – randomly selected from patients receiving a CT/CBCT scan due to suspected pathologies and/or anatomic considerations in connection with impacted tooth.

### Statistical analysis

Several descriptive statistics were calculated for sample I and II: mean and standard deviation for metric variables (i.e., age); absolute and relative frequencies for categorical variables as well as for some of their combinations (i.e., gender, diagnosis of ARR, tooth type/position, location, angulation, region). Further, two logistic mixed models were computed [26]. One model to predict the occurrence of ARR using as fixed factors gender, age, jaw, tooth type/position, and number of impacted teeth (of the patient). For this model, Tukey-type post-hoc tests were calculated for comparison of the various tooth types/positions. In addition, this model was repeated for maxillary canines only using as fixed factors gender, age, and number of impacted teeth (of the patient). The second model predicted the part of the tooth affected by ARR (i.e., either at the crown or root); for this model, all teeth with ARR on both the crown and the root were excluded. This model used as fixed factors gender, age, jaw, tooth type/position, and location. Further, this model was also repeated for maxillary canines only using as fixed factors gender, age, and location. All models included a random effect for the patient, to respect the dependence structure of the data set. For degrees of freedom the Kenward-Roger [27] approximation was used. These models were additionally used to calculate the predicted risk for some specific clinical scenarios. To quantify inter-rater reliability, Cohen’s kappa [28] was computed for the diagnosis of ARR, i.e., a second experienced observer analyzed 50 randomly picked CT/CBCT images. All computations were done using R version 4.3.0 [29].

## Results

### Patient selection process

**Fig. 2:**
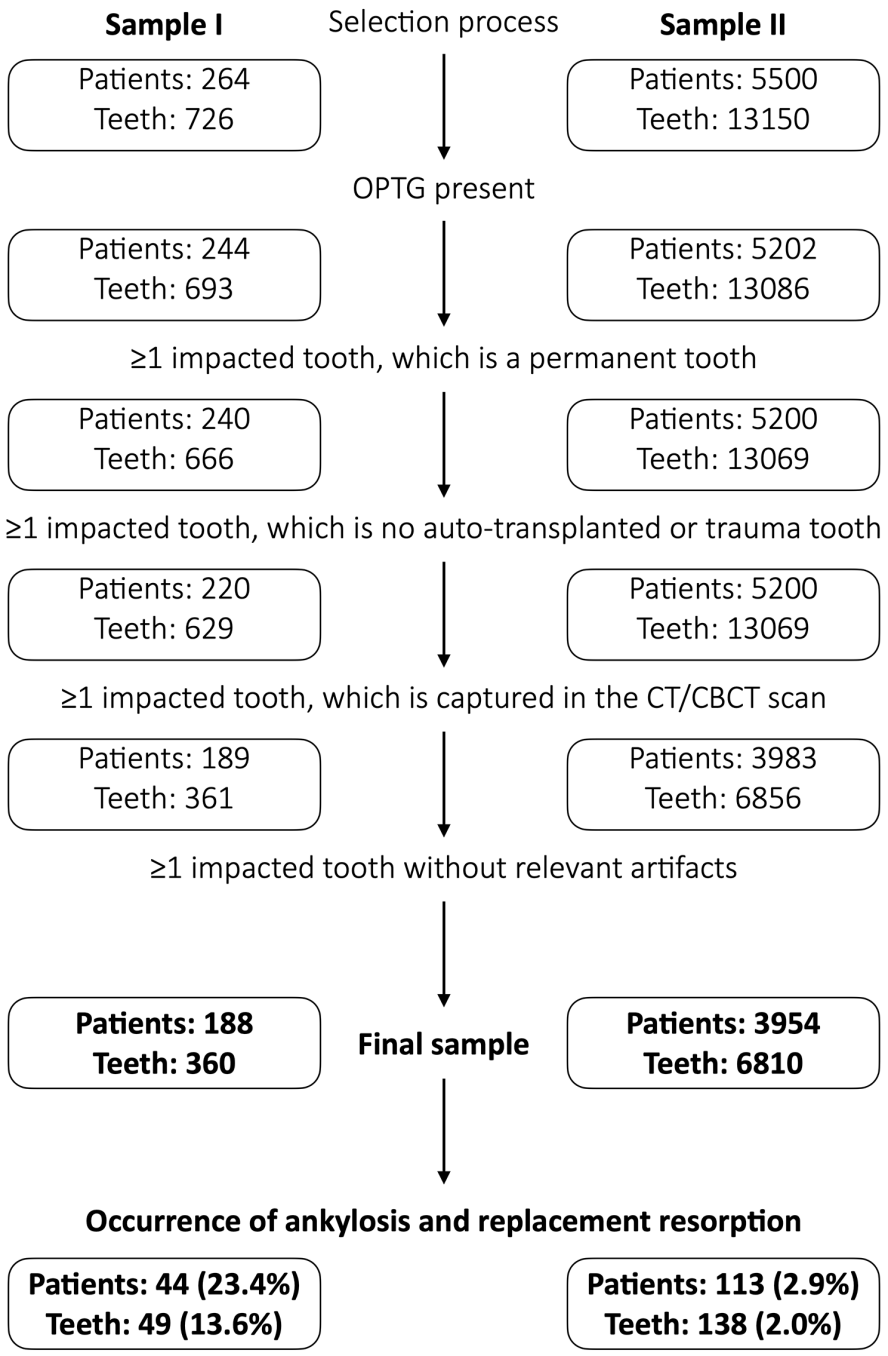
Flowchart of the patient selection process

Sample I included 264 patients (693 teeth); after applying the exclusion criteria 188 patients with at least one impacted tooth being displayed in the CT/CBCT scan were included. In total, sample I consisted of 360 impacted teeth, i.e., those teeth, which had been referred due to suspected dental ARR, as well as all other impacted teeth additionally displayed in the same CT/CBCT scan. For sample II 5500 patients (13,150 teeth) were screened and 3954 patients with at least one impacted tooth being displayed in the CT/CBCT (in total 6810 impacted teeth) were included. Altogether, the present population included 4142 patients with 7170 impacted teeth, which were screened for the presence of ARR (Fig. 2).

### Characteristics of the included patient population and the impacted teeth

Patient age ranged from 5 to 95 years (mean: 31 years) and 57% were female. ARR was detected at 187 impacted teeth (2.6%) of 157 patients (3.7%); 58% of these patients were female. The number of teeth with ARR per patient ranged from 1 to 10 teeth and ARR was about 7-times more frequently diagnosed in sample I compared to sample II, i.e., 13.6 versus 2.0%, respectively.

**Fig. 3:**
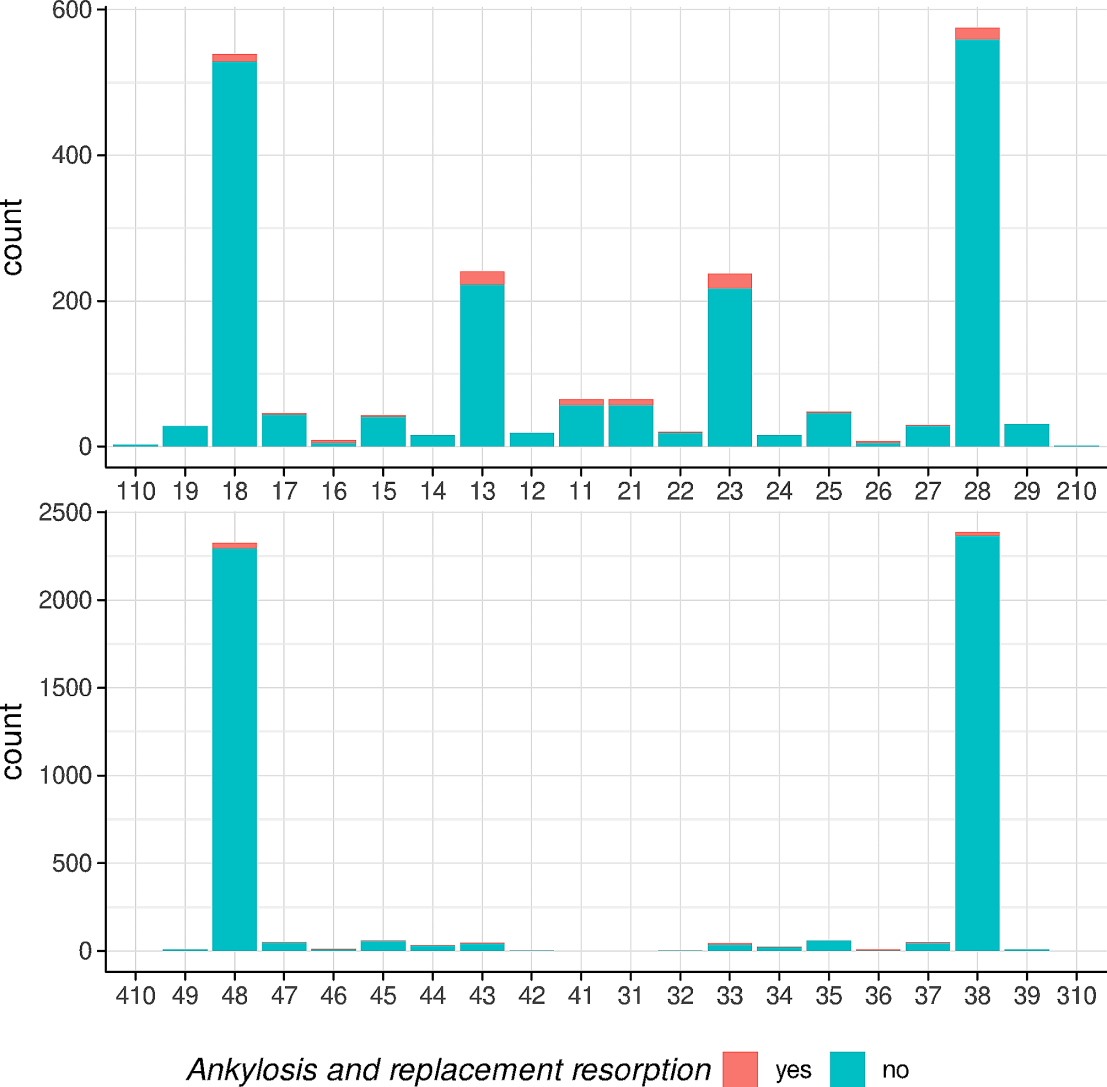
Bar graph presenting the total number of impacted teeth with and without ARR per tooth type and jaw

The distribution of all impacted teeth as well as of those diagnosed with ARR is presented in Table 1; Fig. 3. Overall, 71.5% of the impacted teeth were in the mandible with 1.7% with ARR, while in the maxilla 4.9% of the impacted teeth presented with ARR. The third molars were most often impacted representing 81.3% of the sample, but only 1.4% of them showed signs of ARR. They were followed by the canines (i.e., 7.9% of the sample with 9.3% with ARR) and by the second premolars (i.e., 2.9% of the sample with 2.8% with ARR). Considering the relative frequency distribution, the first molars were most often affected by ARR with a rate of 36.1% (i.e., 13 out of 36 impacted first molars).

The inter-rater reliability (Cohen`s kappa) for the diagnosis of ARR between the first and second observer was 0.726, corresponding to substantial agreement according to Landis and Koch [30].

**Table 1:** Number of impacted teeth and those diagnosed with ARR (count and percentage); the upper and lower jaw, each tooth type as well as “All teeth”, “Sample I”, and “Sample II” are listed separately

		All teeth			Sample I			Sample II		
Jaw	Tooth type	Impacted teeth	Teeth with ARR		Impacted teeth	Teeth with ARR		Impacted teeth	Teeth with ARR	
		***n***	***n***	***%*** ^*1*^	***n***	***n***	***%*** ^*1*^	***n***	***n***	***%*** ^*1*^
**Upper**	***1***	130	16	12.3	22	4	18.2	108	12	11.1
	***2***	39	1	2.6	10	1	10.0	29	0	0
	***3***	479	40	8.4	79	11	13.9	400	29	7.2
	***4***	32	0	0	4	0	0	28	0	0
	***5***	91	4	4.4	17	2	11.8	74	2	2.7
	***6***	17	7	41.2	10	6	60.0	7	1	14.3
	***7***	76	4	5.3	24	0	0	52	4	7.7
	***8***	1114	27	2.4	59	8	13.6	1055	19	1.8
	***9***	59	0	0	0	0	0	59	0	0
	***10***	4	0	0	0	0	0	4	0	0
**Lower**	***1***	2	0	0	0	0	0	2	0	0
	***2***	8	0	0	0	0	0	8	0	0
	***3***	91	13	14.3	15	3	20.0	76	10	13.2
	***4***	56	3	5.4	9	1	11.1	47	2	4.3
	***5***	122	2	1.6	14	0	0	108	2	1.9
	***6***	19	6	31.6	8	3	37.5	11	3	27.3
	***7***	102	9	8.8	21	4	19.0	81	5	6.2
	***8***	4714	55	1.2	67	6	9.0	4647	49	1.1
	***9***	15	0	0	1	0	0	14	0	0
	***10***	0	0	0	0	0	0	0	0	0

^1^Percentages relate to the number of impacted teeth of the specific tooth type/position

ARR, ankylosis and replacement resorption

### Characteristics of the impacted teeth diagnosed with ARR

The characteristics of the 187 impacted teeth diagnosed with ARR are summarized in Table 2. Four out of 5 teeth with ARR (i.e., 78.6%) had a central location in reference to the dental arch. This is mostly due to frequent central location of the first/second molars and wisdom teeth (i.e., 96.2 and 93.9%, respectively), while only about every second incisor and canine had a central location (i.e., 58.8 and 52.8%, respectively). Incisors and canines had frequently also a lingual/palatal location, i.e., in 23.5 and 34.0% of the cases, respectively. Regarding the angulation, the teeth with ARR where mostly either judged as vertically (41.7%) or mesioangularly (37.4%) angulated. Considering the different tooth types, a few variations can be observed. For example, premolars were about 4- to 5-times more frequently distoangularly and palatally/lingually angulated compared to the other tooth types. Further, incisors where about 4- to 6-times more often horizontally angulated compared to the other tooth types, while first/second molars were in 88.5% vertically angulated. Finally, the frequent mesioangular position is mostly due to canines (56.6%) and wisdom teeth (42.7%). For more than half of the teeth (i.e., 57.2%) ARR was diagnosed at the crown, while only about 10.7% were affected at the crown and at the root. Of the teeth with ARR at the root (i.e., 32.1%), the cervical region was most frequently affected (i.e., in about 80.6 and 62.8% of teeth in the maxilla and mandible, respectively), while the apical region was least often affected (i.e., in about 16.7 and 18.6% of teeth in the maxilla and mandible, respectively). Only 6 teeth were diagnosed with ARR extending over the entire root.

**Table 2:** Characteristics of the impacted teeth diagnosed with ARR.

Parameter	Jaw	Subcategories	Tooth type									
			Incisors		Canines		Premolars		1st & 2nd molars		3rd molars	
			*n*	*%* ^*1*^	*n*	*%* ^*1*^	*n*	*%* ^*1*^	*n*	*%* ^*1*^	*n*	*%* ^*1*^
**Location of the tooth in reference to the dental arch**	***Upper***	*central*	10	58.8	17	22.2	2	22.2	11	42.3	24	29.3
		*buccal*	3	17.6	5	9.4	0	0	0	0	3	3.7
		*lingual/palatal*	4	23.5	18	34.0	2	22.2	0	0	0	0
	***Lower***	*central*	0	0	11	20.8	5	55.6	14	53.8	53	64.6
		*buccal*	0	0	2	3.8	0	0	0	0	2	2.4
		*lingual/palatal*	0	0	0	0	0	0	1	3.8	0	0
**Angulation of the tooth**	***Upper***	*buccal*	3	17.6	4	7.5	0	0	0	0	4	4.9
		*distoangular*	1	5.9	0	0	1	11.1	0	0	2	2.4
		*horizontal*	2	11.8	0	0	0	0	0	0	3	3.7
		*mesioangular*	3	17.6	27	50.9	0	0	0	0	4	4.9
		*lingual/palatal*	1	5.9	1	1.9	1	11.1	0	0	2	2.4
		*vertical*	7	41.2	8	15.1	2	22.2	11	42.3	12	14.6
	***Lower***	*buccal*	0	0	0	0	0	0	0	0	2	2.4
		*distoangular*	0	0	2	3.8	1	11.1	1	3.8	2	2.4
		*horizontal*	0	0	1	1.9	0	0	0	0	0	0
		*mesioangular*	0	0	3	5.7	1	11.1	1	3.8	31	37.8
		*palatal/lingual*	0	0	0	0	1	11.1	1	3.8	3	3.7
		*vertical*	0	0	7	13.2	2	22.2	12	46.2	17	20.7
**Part of the tooth with ARR**	***Upper***	*crown*	9	52.9	26	49.1	1	11.1	4	15.4	23	28.0
		*root*	5	29.4	12	22.6	1	11.1	6	23.1	2	2.4
		*crown & root*	3	17.6	2	3.8	2	22.2	1	3.8	2	2.4
	***Lower***	*crown*	0	0	6	11.3	4	44.4	4	15.4	30	36.6
		*root*	0	0	5	9.4	0	0	8	30.8	20	24.4
		*crown & root*	0	0	2	3.8	1	11.1	3	11.5	4	4.9
**Part of the root with ARR** ^2^	***Upper***	*furcation*	0	0	0	0	0	0	4	22.2	2	7.1
		*apical*	3	37.5	3	14.3	0	0	0	0	0	0
		*middle*	6	75.0	5	23.8	2	50.0	3	16.7	2	7.1
		*cervical*	8	100	10	47.6	3	75.0	5	27.8	3	10.7
		*entire root*	3	37.5	1	4.8	0	0	0	0	0	0
	***Lower***	*furcation*	0	0	0	0	0	0	5	27.8	7	25.0
		*apical*	0	0	1	4.8	0	0	1	5.6	6	21.4
		*middle*	0	0	0	0	1	25.0	3	16.7	9	32.1
		*cervical*	0	0	6	28.6	1	25.0	6	33.3	14	50.0
		*entire root*	0	0	0	0	0	0	0	0	2	7.1

^1^ Percentages relate to the whole mouth, i.e., upper and lower jaw combined

^2^*Note* that multiple answers were possible

ARR, ankylosis and replacement resorption; S.D., standard deviation

### Factors associated with ARR

Based on a logistic mixed model any possible effect of gender, age, jaw, tooth type/position, and number of impacted teeth on the occurrence of ARR was analyzed (Table 3). The parameters age and tooth type were significantly associated with ARR. More specifically, higher age was significantly associated with higher odds for ARR (*p* < 0.001), and incisors, canines, premolars, and first/second molars had all higher odds for ARR compared to third molars (*p* < 0.001). The association of tooth type on the occurrence of ARR was further tested in post-hoc tests to allow direct comparisons between all tooth types (Table 3). Incisors as well as first/second molars had significantly higher odds for ARR compared to canines and premolars, respectively, while there was neither a significant difference between incisors and first/second molars nor between canines and premolars.

**Table 3:** Results of a logistic mixed model on factors associated with ARR; OR > 1 indicates a higher chance of ARR than in the reference group

Variable	Level	OR	CI_2.5_	CI_97.5_	z	*p*-value
Logistic mixed model						
*Intercept*		*0.001*	*0.00*	*0.002*	*-20.74*	*< 0.001*
**Gender**	*male*	*Ref.*				
	*female*	1.02	0.74	1.41	0.12	0.904
**Age in decades**		**2.13**	**1.94**	**2.33**	**16.28**	**< 0.001**
**Jaw**	*upper*	*Ref.*				
	*lower*	0.87	0.60	1.28	-0.71	0.478
**Tooth type**	*3rd molars*	*Ref.*				
	*incisors*	**32.04**	**15.85**	**63.25**	**9.86**	**< 0.001**
	*canines*	**6.98**	**4.42**	**11.02**	**8.34**	**< 0.001**
	*premolars*	**3.89**	**1.73**	**7.86**	**3.55**	**< 0.001**
	*1st & 2nd molars*	**25.15**	**14.36**	**43.30**	**11.49**	**< 0.001**
**Number of impacted teeth**		0.99	0.90	1.08	-0.22	0.824

Post-Hoc Tests for Tooth Type						
**Incisors − 3rd molars**		**32.03**	**12.39**	**82.82**	**9.86**	**< 0.001**
**Canines − 3rd molars**		**6.98**	**3.72**	**13.10**	**8.34**	**< 0.001**
**Premolars − 3rd molars**		**3.89**	**1.38**	**10.94**	**3.55**	**0.003**
**1st & 2nd molars − 3rd molars**		**25.15**	**11.78**	**53.70**	**11.49**	**< 0.001**
**Canines - Incisors**		**0.22**	**0.09**	**0.55**	**-4.42**	**< 0.001**
**Premolars - Incisors**		**0.12**	**0.03**	**0.45**	**-4.36**	**< 0.001**
**1st & 2nd molars - Incisors**		0.79	0.28	2.19	-0.64	0.967
**Premolars - Canines**		0.56	0.18	1.71	-1.41	0.606
**1st & 2nd molars - Canines**		**3.60**	**1.54**	**8.43**	**4.07**	**< 0.001**
**1st & 2nd molars - Premolars**		**6.46**	**1.97**	**21.16**	**4.25**	**< 0.001**

Bold values indicate statistical significance

CI, confidence interval; OR, odds ratio; Ref., reference group

The separate analysis for maxillary canines only (Table 4a) showed that, as in the whole sample (Table 3), age was significantly associated with ARR, while the number of impacted teeth was not. Further, female gender was more strongly associated with ARR, albeit still not statistically significant (*p* = 0.063).

**Table 4:** Models including maxillary canines only. (a) logistic mixed model on factors associated with ARR; OR > 1 indicates a higher chance of ARR than in the reference group. (b) logistic mixed model for the part of the tooth with ARR being the root (compared to the crown); OR > 1 indicates a higher chance of ARR at the root (compared to the crown) than in the reference group

Variable	Level	OR	CI_2.5_	CI_97.5_	z	*p*-value
a) Occurrence of ARR (absent vs. present)						
*Intercept*		*0.01*	*0.00*	*0.04*	*-7.89*	*< 0.001*
**Gender**	*male*	Ref.				
	*female*	2.11	0.99	4.89	1.86	0.063
**Age in decades**		**1.50**	**1.28**	**1.77**	**4.95**	**< 0.001**
**Number of impacted teeth**		0.97	0.77	1.18	-0.24	0.813

b) Part of the tooth with ARR						
*Intercept*		*0.22*	*0.01*	*8.63*	*-0.88*	*0.381*
**Gender**	*male*	Ref.				
	*female*	0.11	0.00	1.22	-1.60	0.111
**Age in decades**		**2.28**	**1.33**	**4.83**	**2.59**	**0.009**
**Location**	*central*	Ref.				
	*buccal*	2.41	0.14	56.14	0.60	0.547
	*lingual/palatal*	3.12	0.39	32.35	1.05	0.296

Bold values indicate statistical significance

ARR, ankylosis and replacement resorption; CI, confidence interval; OR, odds ratio; Ref., reference group

### Factors associated with the part of the tooth with ARR

Based on a logistic mixed model any possible effect of gender, age, jaw, tooth type/position, and tooth location on the part of the tooth with ARR (i.e., root versus crown) was analyzed (Table 5). The parameters gender and age were significantly associated with the part of the tooth with ARR. More specifically, female compared to male patients had significantly less often ARR at the root (*p* = 0.009). Further, with increasing age the root was significantly more often affected by ARR than the crown (*p* < 0.001). The other parameters did not significantly affect the specific part of the tooth with ARR. A smaller model for upper canines only yielded similar results (Table 4b).

**Table 5:** Results of a logistic mixed model for the part of the tooth with ARR being the root (compared to the crown); OR > 1 indicates a higher chance of ARR at the root (compared to the crown) than in the reference group

Variable	Level	OR	CI_2.5_	CI_97.5_	z	*p*-value
*Intercept*		*0.09*	*0.01*	*0.50*	*-2.65*	*0.008*
**Gender**	*male*	Ref.				
	*female*	**0.34**	**0.15**	**0.75**	**-2.61**	**0.009**
**Age in decades**		**2.07**	**1.58**	**2.80**	**5.04**	**< 0.001**
**Jaw**	*upper*	Ref.				
	*lower*	0.55	0.22	1.33	-1.3	0.194
**Tooth type**	*3rd molars*	Ref.				
	*incisors*	5.82	1.03	37.75	1.93	0.054
	*canines*	1.97	0.62	6.62	1.13	0.259
	*premolars*	1.16	0.20	7.57	0.17	0.867
	*1st & 2nd molars*	1.53	0.39	6.42	0.60	0.548
**Location**	*central*	Ref.				
	*buccal*	4.03	0.88	22.47	1.71	0.087
	*lingual/palatal*	1.87	0.47	8.00	0.88	0.382

Bold values indicate statistical significance

CI, confidence interval; OR, odds ratio; Ref., reference group

### Predicted risk of ARR at impacted teeth

The predicted risk of ARR at impacted teeth is presented in Table 6. The data are displayed for female patients with one impacted tooth, but separately for the significant parameters in Table 3 (i.e., age and tooth type) and jaw type. The highest predicted risk was recorded for 40-year-old patients with impacted incisors (35.5%) or first/second molars (30.1%) in the upper jaw; this risk was only slightly smaller in the lower jaw, i.e., 32.4 and 27.3%, respectively. While for younger patients (20 years of age) the predicted risk was only about one third for the same tooth types, i.e., about 10 and 8% for incisors and first/second molars, respectively, irrespective of jaw type. Third molars showed the lowest risk (i.e., 0.3 to 1.7%), while canines and premolars ranged between 2.3 and 10.7% and 1.3 to 6.3%, respectively. Data for male patients and/or patients having several impacted teeth are not presented due to only very minor differences but are available upon reasonable request.

**Table 6:** Predicted risk of ARR at impacted teeth for female patients with one impacted tooth, but separately for the significant parameters in table 3 (i.e., age and tooth type) and jaw type

Age	Tooth type	Predicted risk (%)
Upper jaw		
20 years	incisors	10.83
	canines	2.58
	premolars	1.45
	1st & 2nd molars	8.71
	3rd molars	0.38
40 years	incisors	35.46
	canines	10.70
	premolars	6.26
	1st & 2nd molars	30.14
	3rd molars	1.69

Lower jaw		
20 years	incisors	9.58
	canines	2.26
	premolars	1.27
	1st & 2nd molars	7.68
	3rd molars	0.33
40 years	incisors	32.39
	canines	9.45
	premolars	5.50
	1st & 2nd molars	27.34
	3rd molars	1.47

## Discussion

The present retrospective radiographic study analyzed the prevalence and characteristics of ARR at impacted teeth using CT/CBCT scans of more than 4000 patients with 7170 impacted teeth. Only 2.6% of the teeth were impacted in 3.7% of all patients, with incisors and first/second molars having the highest odds for ARR, and higher age significantly increased the odds for ARR. Hence, ARR affecting impacted teeth is indeed a rare condition, but its diagnosis is crucial prior to initiating orthodontic therapy, as it might alter the course and success rate of therapy. Specifically, ARR can be associated with a failure of alignment of the affected tooth and with inhibition of alveolar bone growth [15]. Therefore, CT/CBCT-based treatment planning in cases with impacted teeth is advisable to exclude the presence of ARR and enable the best possible treatment decisions. The possibility of a CT/CBCT examination, however, may not be readily available in many parts of the world. Therefore, understanding which factors are associated with ARR and how high approximately the risk of ARR is at impacted teeth in different clinical scenarios, may be helpful during treatment planning.

To the best of our knowledge, the present study investigated the so far largest pool of patients with impacted teeth of all types, based on CT/CBCT scans. For comparison, previous studies on the prevalence of and factors associated with ARR at impacted teeth focused primarily on maxillary canines with sample sizes ranging from 30 to 225 teeth [13, 15–19, 21], while only two studies investigated so far a larger sample but based on 2-dimensional radiographic diagnostic (i.e., OPTGs) [31, 32]. Depending on the tooth type the prevalence of ARR ranged from 0 (i.e., upper first premolars, lower central and lateral incisors, upper and lower fourth and fifth molars) to 41.2% (i.e., upper first molars), with 4 tooth types presenting a prevalence > 10% (i.e., upper central incisors, lower canines, upper and lower first molars) followed by upper canines and lower second molars with about 8–9% prevalence rate. It should be noted that these prevalence rates are neither representative for a general population nor for a population with impacted teeth. The present sample included only impacted teeth with a medical reason for CT/CBCT recording and, in addition, all patients with suspected ARR were intentionally added to the sample. This leads most likely to an overestimation of the prevalence and thereby also of the predicted risk of ARR compared to any randomly chosen population with impacted teeth. This is also underlined by the comparison of the prevalence rates of sample I and II, i.e., ARR was about 7-times more frequently diagnosed in the sample with suspected ARR compared to the randomly chosen sample (i.e., 13.6 versus 2.0%, respectively). At the same time, it should be noted, that deciduous teeth, auto-transplanted teeth, and teeth after trauma treatment were explicitly excluded to avoid mixing ARR at impacted teeth with ARR occurring frequently due to other reasons.

A direct comparison with previous studies analyzing the prevalence of ARR among impacted teeth is difficult, as there are in general few studies on this topic and those available present often relevant differences in terms of investigated patient sample, case definition, and study design. For example, two previous study with a sample size exceeding 1000 patients reported a comparable to slightly lower rate of ARR [31, 32]. However, the judgement in both studies was based on OPTGs, which is known to be less precise than CT/CBCT scans [25], and the definition of ankylosis was based more on clinical signs and differed therefore from the definition used herein. Another study using CT/CBCT data [20] screened CBCT images of 735 (partially) impacted teeth but selected only those with suspected ARR in the screening process (*n* = 206) and 57 teeth (i.e., 27.7%) were classified as presenting with ARR, which is about twice the prevalence of our sample I (i.e., referral due to suspected ARR). However, this comes without surprise, as herein the “suspicion for ARR” was based on clinical examination and/or 2-dimensial radiographs, while in Rege et al. [20] the selection was based on screening CBCT images. Further, compared to our sample I, the sample of Rege et al. [20] presented differences in the tooth type distribution, i.e., while in the present sample I, canines, first/second molars, and third molars represented about 26–29% each of the teeth with ARR, about half of the teeth with ARR in Rege et al. were canines followed by premolars.

Beside the prevalence rate in different tooth types, also other characteristics of the teeth with ARR were investigated herein. Specifically, ARR occurred almost 3-times more often in the maxilla compared to the mandible, most of the teeth with ARR were located centrally in reference to the dental arch (i.e., about 79%), and about 42 and 37%, respectively, were vertically and mesially angulated. This corresponds overall well to previous data. For example, a more frequent occurrence in the maxilla (about 3.4-times) and mostly vertical (about 23%) and mesioangular (about 49%) angulation has been reported [20]. Interestingly, herein the crown of the impacted teeth was diagnosed quite often with ARR (i.e., in 57.2% of the cases), while this was reported only in 2 cases in Rege et al. [20]; otherwise, both populations (i.e., herein and Rege et al.) showed for ARR at the root high odds to occur cervical, but low odds to occur apical.

In this context, it is of interest whether patient- or tooth-specific parameters affect the occurrence of ARR per se or the part of the tooth affected by ARR, i.e., the crown or root. Based on the present large sample, it was shown that age and tooth type significantly affected the occurrence of ARR, while gender, jaw type, and total number of impacted teeth per patient did not. Specifically, a higher age was associated with higher odds for ARR, and third molars appeared to have the lowest and incisors and first/second molars the highest odds. This is also underlined by the calculation of the predicted risk; for example, 20 years higher age approximately tripled the risk for ARR at impacted teeth. One of the few studies performing a similar analysis [20] showed for some of the parameters similar results (i.e., higher prevalence among anterior teeth and lack of an effect of gender), while other parameters presented different results, at least in terms of statistical significance (i.e., lack of an effect versus higher prevalence in the maxilla, higher prevalence with higher age versus lack of an effect of age). These differences are probably – at least partly – based on the different samples used for the statistical models, i.e., while herein sample I and II were combined, Rege et al. [20] focused on a sample comparable only to the present sample I. Finally, regarding the part of the tooth with ARR, only gender and age were significantly associated, i.e., female compared to male patients had significantly less often ARR at the root, and with increasing age the root was significantly more often affected by ARR than the crown.

As mentioned above, previous studies were focusing often on canines only. After the wisdom teeth, the maxillary canines represented herein the largest sample of impacted teeth, i.e., 479 teeth with 8.4% with ARR. This prevalence rate is overall comparable to previous results. Specifically, in previous studies mostly based on impacted maxillary canines, the occurrence of ARR was for example 6.8% out of 162 teeth [17] or 7% out of 157 teeth [13]. However, also higher prevalence rates of ARR at impacted maxillary canines have been reported, e.g., 20 [15] to 32% [19], which might at least partly be due to patient selection. For example, Becker et al. [15] specifically examined 37 impacted maxillary canines after orthodontic treatment failure. Regarding potentially factors associated with ARR specifically at maxillary canines herein as well as previously [13] a higher age was found to increase the odds for ARR per se, but also for ARR at the root as opposed to the crown.

Obviously, the present retrospective study has besides the above already mentioned selection bias some inevitable limitations, such as the study design per se, which does not allow any conclusions on causality for the development and occurrence of ARR. Further, although having examined a significant number of cases in total, the intended sample size was marginally missed by 127 patients, which is corresponding to approximately 3.1% of the total sample, and the number of cases with ARR is still limited for specific tooth types. Latter limited the flexibility of the applied models and imposes a certain imprecision in the calculated predicted risks. In addition, the diagnostic ability of CT scans might be impeded especially for small defects in the apical third of the root [33]. Nevertheless, CT/CBCT scans are considered as the gold standard in the diagnosis of dental resorptions [34, 35] and are overall well comparable to a histological assessment, while OPTG was judged as not reliable for diagnosis of ARR [25]. Finally, due to our primary aim of assessing a large sample size, a detailed consideration of dental history, previously experienced dental treatment, differentiation between primary failure of eruption [36] and tooth impaction, etc. was not feasible.

In conclusion, the results of the present study confirmed the rare occurrence of ARR at impacted teeth, i.e., only 2.6% of 7170 impacted teeth analyzed with CT/CBCT scans were affected by ARR. On the patient level, higher age significantly increased the odds for ARR and on the tooth level, incisors and first/second molars had the highest odds for ARR, while wisdom teeth had the lowest. For a 40-year-old patient, one can assume that approximately 1 out of 3 impacted incisors or first/second molars might be affected by ARR, whereas for a 20-year-old patient this drops below 1 out of 10. Hence, these results are helpful for orthodontic treatment planning of patients at different age groups, i.e., for younger patients with impacted teeth probably a more conservative and less invasive treatment can be chosen due to lower odds for ARR.

## Data Availability

The datasets used and/or analysed during the current study are available from the corresponding author on reasonable request.

## Appendix 1

STROBE Statement—Checklist of items that should be included in reports of cross-sectional studies item

**Table:**

	Item No	Recommendation
**Title and abstract**	1	(*a*) Indicate the study’s design with a commonly used term in the title or the abstract
		(*b*) Provide in the abstract an informative and balanced summary of what was done and what was found
Introduction		
Background/rationale	2	Explain the scientific background and rationale for the investigation being reported
Objectives	3	State specific objectives, including any prespecified hypotheses
Methods		
Study design	4	Present key elements of study design early in the paper
Setting	5	Describe the setting, locations, and relevant dates, including periods of recruitment, exposure, follow-up, and data collection
Participants	6	(*a*) Give the eligibility criteria, and the sources and methods of selection of participants
Variables	7	Clearly define all outcomes, exposures, predictors, potential confounders, and effect modifiers. Give diagnostic criteria, if applicable
Data sources/ measurement	8	For each variable of interest, give sources of data and details of methods of assessment (measurement). Describe comparability of assessment methods if there is more than one group
Bias	9	Describe any efforts to address potential sources of bias
Study size	10	Explain how the study size was arrived at
Quantitative variables	11	Explain how quantitative variables were handled in the analyses. If applicable, describe which groupings were chosen and why
Statistical methods	12	(*a*) Describe all statistical methods, including those used to control for confounding
		(*b*) Describe any methods used to examine subgroups and interactions
		(*c*) Explain how missing data were addressed
		(*d*) If applicable, describe analytical methods taking account of sampling strategy
		(*e*) Describe any sensitivity analyses
Results		
Participants	13	(a) Report numbers of individuals at each stage of study—eg numbers potentially eligible, examined for eligibility, confirmed eligible, included in the study, completing follow-up, and analysed
		(b) Give reasons for non-participation at each stage
		(c) Consider use of a flow diagram
Descriptive data	14	(a) Give characteristics of study participants (e.g. demographic, clinical, social) and information on exposures and potential confounders
		(b) Indicate number of participants with missing data for each variable of interest
Outcome data	15	Report numbers of outcome events or summary measures
Main results	16	(*a*) Give unadjusted estimates and, if applicable, confounder-adjusted estimates and their precision (e.g., 95% confidence interval). Make clear which confounders were adjusted for and why they were included
		(*b*) Report category boundaries when continuous variables were categorized
		(*c*) If relevant, consider translating estimates of relative risk into absolute risk for a meaningful time period
Other analyses	17	Report other analyses done—eg analyses of subgroups and interactions, and sensitivity analyses
Discussion		
Key results	18	Summarise key results with reference to study objectives
Limitations	19	Discuss limitations of the study, taking into account sources of potential bias or imprecision. Discuss both direction and magnitude of any potential bias
Interpretation	20	Give a cautious overall interpretation of results considering objectives, limitations, multiplicity of analyses, results from similar studies, and other relevant evidence
Generalisability	21	Discuss the generalisability (external validity) of the study results
Other information		
Funding	22	Give the source of funding and the role of the funders for the present study and, if applicable, for the original study on which the present article is based

## Abbreviations

ARR: ankylosis and replacement resorption
CI: confidence interval
CT: computed tomography
CBCT: cone beam computed tomography
OPTG: orthopantomography
OR: odds ratio
Ref.: Reference group
S.D.: standard deviation
